# Physical activity, healthy lifestyle, and subjective wellbeing in people with type 2 diabetes: testing the efficacy of an exercise program

**DOI:** 10.3389/fragi.2025.1522615

**Published:** 2025-09-10

**Authors:** Luís Cid, Diogo Monteiro, Romeu Mendes, Filipa Cláudio, Teresa Bento, Miguel Jacinto, Nuno Couto, Pedro Duarte-Mendes

**Affiliations:** ^1^ Sport Sciences School of Rio Maior (ESDRM), Santarém Polytechnic University (IPSantarém), Rio Maior, Portugal; ^2^ Research Center in Sports Sciences, Health Sciences and Human Development (CIDESD), Vila Real, Portugal; ^3^ ESECS—Polytechnic University of Leiria, Leiria, Portugal; ^4^ Public Health Unit, Local Health Unit of Trás-os-Montes e Alto Douro, Vila Real, Portugal; ^5^ EPIUnit-ITR, Institute of Public Health of the University of Porto, Porto, Portugal; ^6^ Portuguese Directorate-General of Health, Lisboa, Portugal; ^7^ Rio Maior Sports Center (DESMOR), Rio Maior, Portugal; ^8^ Department of Sports and Wellbeing, Polytechnic Institute of Castelo Branco (IPCB), Castelo Branco, Portugal; ^9^ Physical Activity and Health Research and Innovation Center (SPRINT), Castelo Branco, Portugal

**Keywords:** physical activity, healthy lifestyle, wellbeing, exercise, type 2 diabetes

## Abstract

**Introduction:**

This work aimed to first validate the Portuguese version of the FANTASTIC questionnaire that allowed the assessment of lifestyle behaviors through a sample comprising 562 Portuguese subjects; second, through a quasi-experimental study, we tested the effectiveness of a physical exercise program designed specifically for people with type 2 diabetes based on subjective wellbeing and lifestyle changes.

**Methods:**

For 9 months, a total of 31 subjects (14 males and 17 females with type 2 diabetes aged between 58 and 79 years) were involved in a physical exercise program of moderate intensity three times per week for 75 min each session; the program included a combination of aerobic, resistance, agility, balance, and flexibility exercises (*Diabetes em Movimento*).

**Results:**

Regarding the first goal of this study, the results show a reliable factorial solution with nine factors and 27 items from the FANTASTIC questionnaire. With regard to the second goal of this study, the results indicate that subjects involved in the exercise program show significantly improved positive affect, satisfaction with life, physical activity, sleep, stress, and nutrition behaviors, in addition to significantly diminished negative affect. However, these results should be interpreted with some caution as our study did not have a control group and the sample was one of convenience, which limits the capacity of inference of the results.

**Conclusion:**

The present results support evidence confirming the positive effects of Physical Exercise through the *Diabetes em Movimento®* program to foster SWB and promote healthier lifestyle behaviors among T2D subjects. Therefore, we intend to conduct further studies in the future to consolidate the findings of the present study.

## Introduction

The adoption and maintenance of a healthy lifestyle based on five domains, namely nutrition, physical activity, sleep, stress management, and substance abuse (WHO, 1999), has been recognized as a priority by the World Health Organization (WHO, 2024b). With regard to the physical activity (PA) behaviors in Europe, the last Eurobarometer on Sport and Physical Activity (European Union, 2022) identified that Europeans are more sedentary; it also acknowledges that the overweight, obesity, stress, and substance abuse (alcohol and tobacco) rates have risen. The survey identified that approximately 46% of Europeans never exercise or engage in PA and that this number has slightly increased from previous years. Additionally, sedentary behaviors like sitting for long periods of time (over 8.5 h per day) have become more common, leading to public health concerns related to chronic conditions like cardiovascular diseases and type 2 diabetes (T2D) that are associated with unhealthy lifestyles. Effectively, low levels of PA and poor nutritional habits characterized by high calorie intake are highly associated with T2D (Amanat et al., 2020). Diabetes is known to negatively impact the quality of life and subjective wellbeing (SWB) of individuals (Fisher et al., 2010; Hajos et al., 2012). Given the physical limitations and emotional burden associated with diabetes (Lloyd et al., 2001), the satisfaction with life (SWL), positive and negative affect, as well as cognitive and affective variables of SWB (Diener et al., 1985) are influenced by this disease. According to available research, individuals affected by T2D have lower perceptions of SWL, lower levels of positive affect (Anderson et al., 2001; Lloyd et al., 2001), and higher levels of negative affect (Fisher et al., 2010) compared to individuals without the disease.

Lately, there has been an increase in T2D prevalence worldwide; nearly 537 million adults have been diagnosed with diabetes, and it is expected that this number will increase to 783 million by 2045 (IDF, 2021). In Portugal, diabetes reportedly affect 13% of the adult population. The IDF (2021) also projects a significant increase in the number of diabetes cases in this country given its large elderly population as well as high prevalence of obesity and sedentary behaviors. Hence, physical exercise (PE) is an essential tool in the management of T2D subjects as it can improve glucose control, potentiate insulin sensitivity, and promote cardiovascular and overall health (Colberg et al., 2016). According to Umpierre et al. (2011), exercise helps lower blood sugar levels by increasing its usage; thus, exercise increases the body’s demand for energy, which the muscles obtain from glucose. PE practice along with dietary changes can improve weight management, which in turn improves blood glucose regulation and insulin sensitivity (Knowler et al., 2002). With regard to exercise typology, research has shown that combining different exercise forms (aerobic and resistance aerobic) could improve some of the glucose metabolism markers and inflammatory parameters in sedentary adults without diabetes (Ferreira et al., 2022; Silva et al., 2024); combination exercises may also be more effective for management of T2D than aerobic or resistance exercises alone (Schwingshackl et al., 2014). Moreover, according to Colberg et al. (2010), exercise programs designed specifically for T2D subjects can improve mobility and aerobic resistance, which could facilitate the daily PAs in these individuals’ routines (e.g., climbing stairs, walking more, or doing household tasks). Adherence to an exercise program can build a virtuous cycle, where the perceived benefits reinforce a physically active lifestyle. Exercise can also contribute to behavioral changes in other areas essential to T2D management. In addition, individuals involved in exercise programs are more aware of the importance of dietary control for optimizing glycemic control. According to Wing and Phelan (2005), exercise practice is often associated with a concern for the diet since people who adopt active lifestyles are also looking for complementary improvements to their nutritional lifestyles.

The benefits of a healthy lifestyle are well-defined and linked to a higher average life expectancy as well as a lower likelihood of contracting serious chronic diseases (Oftedal et al., 2019; Silva et al., 2024); thus, there is emerging interest for researchers to be able to evaluate the lifestyle of the population, which in turn indicates the need to aggregate different lifestyle parameters in a single instrument. To address this need, the *Fantastic Lifestyle* (Wilson et al., 1984) was developed to assess the lifestyle of subjects; this questionnaire comprises nine factors and 25 items that explore the habits and behaviors concerning population lifestyle. This instrument analyzes the physical, psychological, and social behaviors associated with the FANTASTIC acronym, namely, family and friends (F), activity (A; physical activity), nutrition (N), tobacco/toxins (T; tobacco and drugs), alcohol (A), sleep/stress (S), type of personality (T), insight (I), and career (C). The original version of this questionnaire (Wilson et al., 1984) has nine factors and 25 items, where some factors only have two items; this instrument has already been tested in Mexico in people with high blood pressure (López-Carmona et al., 2000) and T2D (Moctezuma et al., 2003). Additionally, Añez et al. (2008) and Ramírez-Vélez Agredo (2012) confirmed that the initial structure of this scale was reliable for accessing the lifestyles of Brazilian and Colombian adults, respectively. In Portugal, Silva et al. (2024) identified that this instrument can be used to assess the lifestyles of Portuguese young adults through a factorial structure comprising nine factors and 30 items; even with some factors having less than three items, this factorial structure is different from the original one proposed by Wilson et al. (1984), which reveals that the questionnaire can have different factorial compositions.

Independently of the above, the presence of factors with only two items is a drawback of the *Fantastic Lifestyle* questionnaire since it is paramount to have factors with at least three items (Kline, 2016). According to Nunnally and Bernstein (1994), factors with only two items may have issues regarding stability, and the reliability may be affected considering that two items are not sufficient for robust measurements with the model. Moreover, factors with few items (i.e., <3) may not be sufficiently developed to capture the full scope of the constructs in question (Worthington and Whittaker, 2006). Beyond these concerns, to the best of our knowledge, previous studies on the psychometric qualities of the *Fantastic Lifestyle* questionnaire were based on exploratory factor analysis (EFA). To validate the internal structures of the scales and confirm the theoretical constructs, it is essential to perform confirmatory factor analysis (CFA) (Cid et al., 2022); this methodology helps ensure that the relationships between the observed variables and their underlying latent factors are consistent with theoretical expectations. Considering that the assessment instruments developed specifically for this area are not abundant or inclusive of some limitations, such as the *Fantastic Lifestyle* questionnaire, researchers have two options for action (Fonseca and Paula Brito, 2005) as follows: (1) develop new instruments; (2) make adaptations of instruments already existing in other languages into Portuguese.

Given the intention of developing a questionnaire with more robust psychometric qualities to assess the lifestyles of Portuguese adults, including those with T2D, and considering the abovementioned concerns, we aimed to develop and analyze the factorial structure of the new Portuguese version of the *Fantastic Lifestyle* questionnaire (nine factors and 28 items) through EFA and CFA in study I. This version is derived from the original and also includes the full Brazilian version (Añez et al., 2008) as well as a part of the Portuguese version (Silva et al., 2014), thereby departing from the original by having one more factor and five more items. This new version includes three items per factor to address the reliability issues in the previous version. Furthermore, considering the issues related to the population’s lifestyle; prevalence of T2D in Portugal and globally; positive impacts of combined exercises on disease management, quality of life, and SWL; as well as promotion of daily PA behaviors and nutritional alterations, we aimed to implement and assess the effects of the *Diabetes em Movimento* ^®^ on the SWB, daily physical activity, and nutritional habits in Portuguese subjects with T2D through study II.

The effects of combining PE programs on the health and SWB of T2D subjects are well defined (Chudyk and Petrella, 2011; Hayashino et al., 2012; Mendes et al., 2016; Oliveira et al., 2012). However, many of these programs require expensive equipment and are difficult to access by subjects with pathologies and older people, which represents a high burden on healthcare and unavailability to most people with T2D (Mendes et al., 2016). Beyond these community PE programs for specific population groups, the WHO has suggested recommended interventions to promote lifestyle changes (Mendes et al., 2017; WHO, 2009); these interventions appear to be cost-effective and more applicable than individual interventions and have also been linked to better adherence and long-term results in the T2D population (Plotnikoff et al., 2013; Sazlina et al., 2013). The main goal of this work was to test the effectiveness of a PE program - *Diabetes em Movimento* (Mendes et al., 2016, 2017) designed specifically for people with T2D on their SWB (i.e., satisfaction with life and different affect) as well as lifestyle changes based on the main dimensions of the PANSAS model that is based on the WHO’s global recommendations for physical activity and health. Furthermore, we propose to validate (in study I) the Portuguese version of the FANTASTIC questionnaire (Wilson et al., 1984) that allows assessment of the various dimensions of lifestyle.

## Study I

### Methods

#### Samples

Under the EFA category, we enrolled 202 subjects including 47 persons with T2D and 155 adults without diabetes. The diabetic subjects were aged between 51 and 79 years (mean (M) = 67.79; standard deviation (SD) = 5.74), while the non-diabetic students with higher education levels were aged between 18 and 38 years (M = 21.85; SD = 3.06). Under the CFA category, we recruited 562 subjects including 67 persons with T2D and 495 non-diabetics; here, the diabetic subjects were aged between 51 and 79 years (M = 68.77; DP = 6.07), while the non-diabetic students with higher education levels were aged between 18 and 37 years (M = 22.87; DP = 3.74).

### Instruments

The adapted versions of the *Fantastic Lifestyle* questionnaire (Wilson et al., 1984) were used in this part of the study, as noted earlier. Considering that good practices recommend a minimum of three items to successfully saturate the latent factor (Hair et al., 2019; Kline, 2016), the adapted version proposed here for the Portuguese population comprised 28 closed-response items that explored the habits and behaviors of a given population in relation to their lifestyle. The subjects responded on a 5-level Likert scale ranging from 1 (i.e., never) to 5 (i.e., always). This instrument analyzes different areas of physical, psychological, and social behaviors associated with the acronym FANTASTIC, as noted earlier.

### Procedures

EFA: Here, the measurements of the central trend (i.e., average) and dispersion (e.g., default deviation) were first analyzed; subsequently, EFA was carried out by considering a ratio of 5:1 (number of subjects per item in the questionnaire) (Hair et al., 2019) based on the recommendations of several authors (Hair et al., 2019; Kline, 2016) as follows: method of main components with oblique rotation; Kaiser criterion (eigenvalue ≥1.0); acceptable Kaiser–Meyer–Olkin criterion (KMO ≥0.08; *p* ≤ 0.001); sampling measurement and Bartlett test for sampler suitability and sphericity; factor weights ≥0.50; variance explained by factors ≥40%; internal consistency (Cronbach’s alpha ≥0.70), where the value should not increase if the item is eliminated; only factors with at least three items should be returned. The EFA was performed using SPPS 25.0 software.

CFA: To validate the *Fantastic Lifestyle* questionnaire, we conducted CFA based on the recommendations of some authors (Byrne, 2016; Hair et al., 2019; Kline, 2016), specifically using the method of maximum likelihood (MML), chi-squared test (χ2), degrees of freedom (df), and level of significance (*p*), along with the following adjustment indices: standard deviation root mean-squared residual (SRMR), comparative fit index (CFI), Tucker–Lewis index (TLI), root mean-squared error of approximation (RMSEA), and their confidence intervals (CIs; 90%). For these indices, the threshold values suggested by some authors (Byrne, 2016; Hair et al., 2019; Kline, 2016) were adopted, namely, SRMR ≤0.80, CFI and TLI ≥0.90, and RMSEA ≤0.80. The convergent validity was calculated to analyze whether the items converged strongly for the factors associated with them via the average variance extracted (AVE) for each factor by considering adjusted values ≥0.50 (Fornell and Larcker, 1981; Hair et al., 2019). Then, the discriminating validity was analyzed to verify whether the factors were sufficiently different from each other; these were considered to be adjusted when the square of the correlations between the factors was not greater than the AVE of each factors, and the internal consistency was calculated using composite reliability (Raykov, 1997) by considering adjusted values ≥0.70 (Hair et al., 2019).

## Results

EFA: From the data presented in Table 1, we observe that nine factors were extracted from EFA with eigenvalue ≥1, justifying overall 57.75% total variance of the results; one item (item 12) that showed cross-loading between the factors tobacco and nutrition was not considered acceptable and was eliminated. Thus, the final analysis included nine factors with 27 items, and the results of the factors were quite acceptable (≥0.50), except for item 12. The values of the factorial weights for the respective factors varied between 0.51 and 0.17, and all items consequently explained at least 25% of the variance of the latent factor, while the Cronbach’s alpha of factors varied between 0.74 and 0.82. From Table 2, we observe that family was the factor with the highest value (3.54 ± 0.51) while sleep/seatbelts/stress was less valued by the subjects (0.72 ± 0.53). The descriptive analysis revealed that the subjects valued factors related to family, nutrition, personality traits, and career more than the others. However, most of the correlations were significant both directly and inversely, with a negative correlation between family and alcohol/addictive substance consumption as well as a positive correlation between introspection and career.

**TABLE 1:** Exploratory factorial analysis of the *Fantastic Lifestyle* questionnaire.

FANTASTIC items	Communalities	F	A	N	T	A	S	TP	I	C
Item 1 (F)	0.63	**0.73**								
Item 2 (F)	0.66	**0.74**								
Item 3 (F)	0.63	**0.70**								
Item 4 (A)	0.63		**0.56**							
Item 5 (A)	0.59		**0.52**							
Item 6 (A)	0.54		**0.64**							
Item 7 (N)	0.68			**0.65**						
Item 8 (N)	0.58			**0.52**						
Item 9 (N)	0.55			**0.64**						
Item 10 (T)	0.54				**0.52**					
Item 11 (T)	0.51				**0.57**					
Item 12 (T)	0.33			0.47	**0.51**					
Item 13 (T)	0.74				**0.62**					
Item 14 (AL)	0.56					**0.59**				
Item 15 (AL)	0.51					**0.53**				
Item 16 (AL)	0.58					**0.51**				
Item 17 (S)	0.54						**0.62**			
Item 18 (S)	0.58						**0.63**			
Item 19 (S)	0.70						**0.54**			
Item 20 (TP)	0.56							**0.66**		
Item 21 (TP)	0.64							**0.72**		
Item 22 (TP)	0.60							**0.72**		
Item 23 (I)	0.56								**0.68**	
Item 24 (I)	0.51								**0.51**	
Item 25 (I)	0.58								**0.59**	
Item 26 (C)	0.58									**0.59**
Item 27 (C)	0.55									**0.65**
Item 28 (C)	0.66									**0.62**
Variance		19.04%	8.85%	6.46%	5.57%	5.01%	4.38%	3.95%	3.58%	3.41%
Cronbach’s alpha		α = 0.74	α = 0.75	α = 0.78	α = 0.71	α = 0.72	α = 0.80	α = 0.76	α = 0.74	α = 0.82
Mean ± Standard deviation		4.04 ± 0.71	3.93 ± 0.81	3.42 ± 0.64	2.77 ± 0.68	2.73 ± 0.48	3.62 ± 0.64	2.53 ± 0.58	2.94 ± 0.38	3.89 ± 0.52

Note: F, family and friends; A, activity; N, nutrition; T, tobacco/toxins; AL, alcohol; S, sleep/stress; TP, type of personality; I, insight; C, career.

**TABLE 2:** Correlation matrix between the factors of the *Fantastic Lifestyle* questionnaire.

Factors	Min–Max	M±SD	1	2	3	4	5	6	7	8	9
1. Family	1–5	3.54 ± 0.51	1	-	-	-	-	-	-	-	-
2. Activity	1–5	0.86 ± 0.56	−0.69**	1	-	-	-	-	-	-	-
3. Nutrition	1–5	3.51 ± 0.53	0.78**	−0.38**	1	-	-	-	-	-	-
4. Tobacco	1–5	1.09 ± 0.51	−0.56**	0.80**	−0.41**	1	-	-	-	-	-
5. Alcohol	1–5	2.98 ± 0.43	−0.91**	−0.67**	0.69**	−0.63**	1	-	-	-	-
6. Sleep/stress	1–5	0.72 ± 0.53	−0.62**	0.84**	−0.36**	0.88**	−0.75**	1	-	-	-
7. Type of personality	1–5	3.17 ± 1.04	0.06	−0.13**	0.05	0.03	0.03	−0.05	1	-	-
8. Insight	1–5	2.92 ± 0.56	0.05	−0.11**	0.08*	−0.10*	0.03	−0.09*	0.65**	1	-
9. Career	1–5	3.18 ± 0.71	0.11*	−18**	0.15**	−0.10*	0.09*	−0.12**	0.78**	0.85**	1

Note: Min–Max = minimum–maximum; M, mean; SD, standard deviation.

CFA: A preliminary analysis of the data was carried out on the missing values as well as univariate and multivariate distributions, as suggested by Hair et al. (2019). The results showed that there were no missing values or violations of the univariate normal distribution since the asymmetry was between −2 and +2 and kurtosis was between −7 and +7 (Byrne, 2016). However, the assumption of multivariate distribution was found to be violated as the multivariate Mardia kurtosis coefficient exceeded the recommended cutoff value (>5) (Byrne, 2016). In this sense, the bootstrap procedure (2,000 samples) was used for subsequent analyses, as suggested by Nevitt and Hancock (2001). The analysis of the *Fantastic Lifestyle* measurement model (nine factors/27 items) fitted the data based on the cutoff values adopted: (χ2 = 556.40; SRMR = 0.035; bootstrapped *p* = <0.001; RMSEA = 0.040 [90% CI = 0.035, 0.045]; TLI = 0.946; CFI = 0.956). We also identified that AVE was ≥0.50 and composite reliability was >0.70 for all the factors, as indicated in Table 3, which consequently explained at least 25% of the latent factor variance. However, some discriminant validity problems were identified involving the factors family and alcohol consumption/addictive substances; physical activity and sleep/stress; nutrition and sleep/stress; and introspection and career, where the squared values of the correlations between the factors were higher than the AVE values of the individual factors. There were no observed problems with the discriminant validity for the remaining factors.

**TABLE 3:** Convergent and discriminant validities as well as composite reliability.

Factors	AVE	CR	1	2	3	4	5	6	7	8	9
1. Family	0.50	0.74	1	-	-	-	-	-	-	-	-
2. Activity	0.52	0.76	0.36	1	-	-	-	-	-	-	-
3. Nutrition	0.60	0.82	0.44	0.09	1	-	-	-	-	-	-
4. Tobacco	0.55	0.79	0.21	0.48	0.13	1	-	-	-	-	-
5. Alcohol	0.59	0.81	0.69	0.32	0.36	0.30	1	-	-	-	-
6. Sleep/stress	0.52	0.78	0.27	0.55	0.08	0.62	0.45	1	-	-	-
7. Type of personality	0.50	0.73	0.002	0.01	0.001	0.001	0.001	0.001	1	-	-
8. Insight	0.51	0.75	0.002	0.01	0.01	0.01	0.001	0.01	0.29	1	-
9. Career	0.51	0.76	0.001	0.02	0.02	0.01	0.01	0.01	0.46	0.56	1

Note: AVE, average variance extracted; CR, composite reliability.

## Discussion

The EFA showed that the subjects valued the factors under analysis and that they all had adequate internal consistency since the Cronbach’s alpha value always exceeded 0.70, which is considered the minimum value for internal validation of an instrument (Hill and Hill, 2008). In the study by Silva et al. (2014), only the overall Cronbach’s alpha was calculated but not the other parameters. Even so, in our analysis, the lowest alpha value is 0.71 and highest is 0.82, which are higher than the values obtained for the preliminary validation (which were below the minimum recommended values). Thus, we observe that all the factors in the final instrument are applicable for their intended measurements. The factor weights were also satisfactory since they were significant and higher than 0.50, thus verifying that all the items explained at least 25% of the variance of the latent factor (Hair et al., 2019). During this analysis, a cross-loading was found, where item 12 was associated with both nutrition and tobacco factors. Considering that the contents of items 11 and 12 are integrated into a single item in the original version and are separated in the Brazilian version of the questionnaire as well as considering that the factor tobacco had four items, we decided to remove item 12 from the final factorial structure as in the study by Dinzeo et al. (2013), as suggested by Kline (2016). However, the total variance of our results was 57.75%, which is higher than the 55.02% reported by Dong et al. (2012) and the 33.33% reported by Dinzeo et al. (2013). The present version (i.e., with 27 items) was built on the basis of theoretical and psychometric criteria by considering that three items were sufficient to represent the latent constructs and warrant model identification in the CFA process, as suggested in previous studies (Worthington and Whittaker, 2006). Based on this initial model, a CFA was conducted as we identified the need to carry out more robust analyses.

In terms of the descriptive analysis, as shown in Table 2, we found that the subjects valued the factors family, nutrition, personality traits, and career more highly, whereas the subjects surveyed by Silva et al. (2014) valued the factors alcohol, personality, introspection, and sleep/stress more highly. This difference can be explained by the fact that the population in the present study is made up of both students and older people while the preliminary validation sample consisted of only university students. During the analysis, most of the correlations between the factors were significant, with the strongest correlations observed being between family and alcohol (−0.91) as well as introspection and career (0.85). Such correlations were not calculated in the preliminary validation (Silva et al., 2014), and only correlations between the factors and overall lifestyle score were analyzed, which showed the strongest correlation between the introspection and personality factors. In the study by Darviri et al. (2014), all the correlations were positive and significant, suggesting that individuals who adopted healthier eating habits and followed an organized routine including PA also sought social and mental support. We also found that there were no convergent validity problems since the AVE from the factors was equal to or greater than 0.50 (Hair et al., 2019), indicating that the items converged strongly for the corresponding factors. The main objective of this work was achieved with the adjustment of the measurement model. However, we are unable to compare these results because the previous versions of the *Fantastic Lifestyle* questionnaire did not report analyses of this nature, as mentioned above. Thus, the present study contributes to the dissemination of knowledge regarding lifestyle and manages to empirically demonstrate the factor structure of this questionnaire, thus addressing an existing gap in literature. In short, the present study shows that this modified questionnaire can be used to generally assess the lifestyle of the Portuguese population. Although the preliminary validation showed some gaps, the more robust analyses that were carried out later showed that the instrument in its current final version reveals good psychometric qualities in terms of internal consistency, convergent and discriminant validities, as well as adequate fit of the measurement model to the data.

Although this study adds to knowledge in this area and helps to fill the gaps identified previously, it has some limitations. First, the sample cohort is composed of only students and people with T2D, so the results cannot be generalized to other populations; this sample was obtained by convenience and comprises two different populations, which can influence the identified factorial structure when considering that different samples may interpret the items differently. Therefore, future studies should use the current instrument and validate it for other segments of the population. Second, considering the sample heterogeneity, it was not possible to carry out an analysis of invariance owing to the different characteristics of the samples under study; in this sense, future studies should try and analyze the invariance of the measurement model based on gender, age, and other sociodemographic characteristics. Third, considering the cross-sectional nature of this study, future studies would need to carry out experimental and/or longitudinal analyses, e.g., through longitudinal invariance.

## Conclusion

Based on the results obtained through EFA and CFA, we show that the present version of the *Fantastic Lifestyle* questionnaire (nine factor and 27 items) is a valid and reliable tool for assessing the lifestyle factors among the Portuguese population.

## Study II

### Methods

#### Samples

This part of the study comprised 31 subjects (14 male and 17 female) with T2D aged between 58 and 79 years (M = 69.11; SD = 5.15). These participants were included in the *Diabetes em Movimento* ^®^ program based on the following inclusion criteria at baseline: more than 6 months after clinical diagnosis of T2D; aged between 50 and 80 years; independent life in the community; registered and followed up in consultation at health units; glycated hemoglobin (HbA1c) levels between 6.0% and 10.0% in the last 6 months; absence of complications from diabetes (e.g., diabetic foot, retinopathy, or nephropathy); absence of symptoms suggestive of coronary artery disease to be clarified (e.g., chest pain, dyspnea, dizziness, or palpitations); absence of serious cardiac, pulmonary, oncological, endocrine, infectious, or musculoskeletal pathologies; no significant changes in gait or balance; non-smoker in the last 6 months; not started insulin therapy or treatment with sulfonylureas in the previous 3 months; residence in the municipality where the exercise program was implemented.

For 9 months, the chosen subjects were involved in the PE program *Diabetes em Movimento* ^®^ developed by Mendes et al. (2017) and registered in the Current Controlled Trials (reference number ISRCTN09240628). This program includes 75-min sessions of moderate intensity (monitored using a heart rate monitor and the Borg Scale) three times a week (Monday, Wednesday, and Friday); the intensity was maintained between 40% and 59% of the heart rate reserve or between 12 and 13 points in the subjective perception effort (on the Borg Scale of 6–20 points) (Borg, 1982). The heart rate monitor was used to continuously assess cardiac frequency during the sessions, which allowed us to ensure that the individuals maintained moderate intensity of effort during each session (i.e., between 40% and 59%) while allowing real-time recording and subsequent data analysis. We also applied the Borg Scale (6–20 points) at the end of each session to complete the intensity control. Although we recognize that post-effort assessment does not guarantee that the effort remained between 12 and 13 during the session, the combination of this methodology with real-time heart rate monitoring and technician orientation allows us to confirm the intensity of exercise during each session. The participants were made familiar with the use of this methodology. The combination of objective and subjective measurements of the effort is well documented in literature (Borg, 1998).

The proposed exercise program was built according to the main international recommendations about PE for T2D, adult, and older adult populations by including a combination of aerobic, resistance, agility, balance, and flexibility exercises (American College of Sports Medicine 2022; Colberg et al., 2010; Mendes et al., 2016; Nelson et al., 2007). The sessions included a group of maximum 40 people and were supervised by a exercise professional nurse; the exercises were performed at sporting facilities with low-cost materials such as chairs, water bottles filled with sand (0.5 kg), gymnastic balls, cones and flares, sticks, and colorful vests. The PE program sessions were designed with the following structure: warm-up (10 min); aerobic exercise (30 min); resistance exercises (20 min) including three exercises each for the upper and lower body performed in a circuit with 20–30 repetitions in 1–4 sets; balance and agility exercises performed through presports and adapted traditional games (10 min); flexibility and return to calm exercise (5 min) as the last phase of the session. There were five different (i.e., A, B, C, D, and E) standardized session plans in the program, each with different aerobic, resistance, and agility/balance exercises. The plans were applied successively over time to induce variability in the stimuli. In addition, health education sessions (once a week) were conducted on the central themes linked to the program, namely PA, nutrition, healthy lifestyle, behavioral change, and diabetes, among others. These sessions were conducted after the PE sessions. To ensure the safety of the T2D individuals enrolled in this program, glycemic control was assessed before and after each PE session by a qualified nurse present at each facility; it is important to note that these data were collected as a security procedure.

#### Instruments

The Portuguese version of the *Fantastic Lifestyle* questionnaire (Wilson et al., 1984) was validated in study I, as noted earlier. This instrument consists of 27 closed-response items exploring the habits and behaviors of a given population in relation to their lifestyle and analyzes the areas of physical, psychological, and social behaviors associated with the FANTASTIC acronym. By taking into consideration the sample specificity of the present study and based on the PANSAS lifestyle factors, only the physical activity, nutrition, and sleep/stress factors were considered.

The Portuguese version of the positive and negative affect schedule (PANAS; Antunes et al., 2020) was also used herein. As suggested by the authors, we used the reduced version containing two factors with five items each, to which the subjects responded on a 5-level Likert scale ranging from 1 (“nothing or very slightly”) to 5 (“extremely”). These factors represent the degrees of positive (enthusiastic, inspired, determined, active, and interested) and negative (frightened, scared, tormented, nervous, and guilty) affect.

The Portuguese version of the satisfaction with life scale (SWLS; Antunes et al., 2019) was administered lastly. This scale contains five items, to which the subjects responded on a 7-level Likert-type scale ranging from 1 (“completely disagree”) to 7 (“completely agree”). Subsequently, the items were grouped into a single factor to represent the overall index of life satisfaction (e.g., “In many fields, my life is close to my ideal.”).

#### Procedures

**FIGURE 1:**
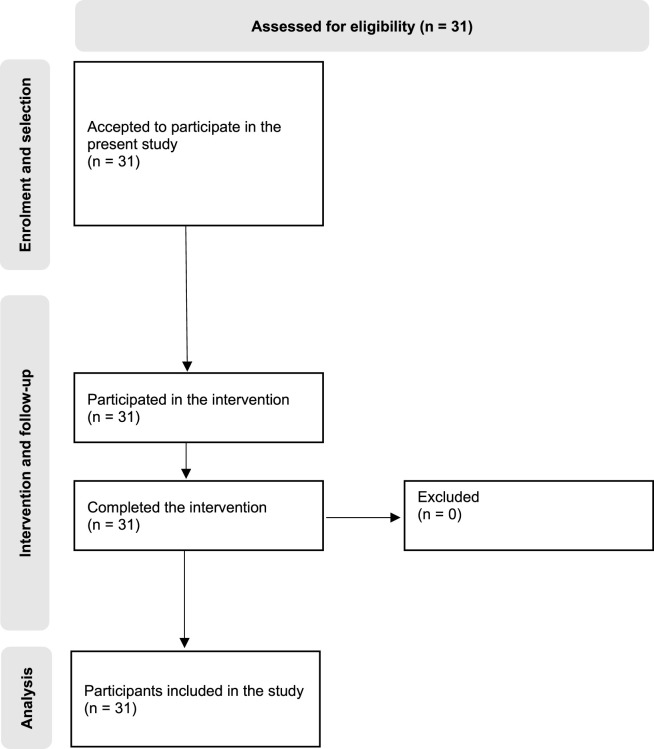
Flowchart showing the participants’ progress through the different phases of the study.

Before conducting the study, the participants were asked to provide informed consent to participate in the interventions. The participants authorized the collection of information for strictly scientific purposes after being guaranteed confidentiality, as stipulated in the guidelines of the Declaration of Helsinki. The instruments were applied individually in a room prior to the exercise sessions and by the exercise technician responsible for the program. For subjects who had difficulty reading or interpreting the information, assistance was provided by research assistants appointed for this purpose during the completion of the questionnaires. The instruments were administered to each participant twice, namely, the beginning (October 2018) and end (July 2019) of the program. The questionnaire completion required approximately 15 min and was offered on the days of the initial and final sessions of the program. A flowchart depicting the participants’ progress through the study procedures is shown in Figure 1.

#### Statistical analyses

Initially, we conducted a descriptive analysis of the study variables using the mean and standard deviation as these variables were considered to be normal; according to the assumptions inherent in the central limit theory, it is safe to assume normality in samples greater than or equal to 30 observations (Hair et al., 2019). To verify the existence of significant differences between the two timepoints in the analysis, a paired t-test (comparison of a subject at the two timepoints) was conducted, assuming *p* ≤ 0.05 as the criterion to reject the null hypothesis (Ho et al., 2007). Additionally, the effect size (Cohen’s d) was calculated by considering the following threshold values: 0.2 for small effect; 0.5 for medium effect; 0.8 for large effect (Cohen, 1988). The data were then analyzed using the statistical software SPSS 26.0 (IBM Corp., Armonk, NY, United States).

## Results

A preliminary analysis of the data revealed that there were no missing values. The sample size was then calculated using the G*Power 3.1 software (Faul et al., 2009), following the recommendations of Denis (2019), and included the following parameters: anticipated effect size of f2 = 0.6, α = 0.05, statistical power = 0.95, and a minimum of 31 subjects. All of these criteria were respected in this study. The exercise program was paused for 1 week each during the Christmas and Easter seasons, and adherence was monitored using a presence register. There was no participant dropout, and the average adherence in each session was 70%. Based on the results shown in Table 4, from a descriptive point of view, the mean values increased in the case of the positive affect, SWL, PA, and nutrition while decreasing for negative affect. From an inferential point of view, the results showed significant differences in all factors from the initial to final sessions (*p* < 0.05) with large effects, revealing that the PE program was important for both SWB and certain lifestyle factors like PA and nutrition.

**TABLE 4:** Descriptive and inferential analyses of the study variables.

Factors	Min–Max	Mean ± SD	*p*	95% confidence interval	d
Positive affect 1	2–5	3.3 ± 0.81	<0.01	[0.351–0.542]	0.51
Positive affect 2	2–5	3.7 ± 0.77			
Negative affect 1	1–4	2.2 ± 0.81	0.04	[0.088–0.308]	0.25
Negative affect 2	1–3	2.0 ± 0.76			
Satisfaction with life 1	3–6	5.1 ± 0.90	0.02	[0.051–0.394]	0.30
Satisfaction with life 2	3–7	5.4 ± 0.1.1			
Physical activity 1	2–5	3.9 ± 0.76	<0.01	[0.355–0.592]	0.55
Physical activity 2	2–5	4.3 ± 0.67			
Nutrition 1	2–4	2.8 ± 0.63	0.04	[0.009–0.353]	0.49
Nutrition 2	2–4	3.1 ± 0.60			
Sleep/Stress 1	2–5	3.6 ± 0.71			
Sleep/Stress 2	2–5	4.0 ± 0.60	<0.001	[0.212–528]	0.61

Note: Min–Max, minimum–maximum; M, mean; SD, standard deviation; 1, baseline; 2, post-intervention; *p*, significance value; d, effect size.

## Discussion

In study II, we aimed to implement and assess the effects of the *Diabetes em Movimento* ^®^ program on SWB, daily PA, and nutritional habits in Portuguese subjects with T2D. The results indicate an increase in the average values from the initial to final phases of the program, with significant differences in all parameters except the values of the negative affect, which decreased considerably. This means that the people involved in the *Diabetes em Movimento* ^®^ program reported improved positive indicators of SWB, PA, and nutritional habits as well as diminished negative affect. These results are very encouraging and highlight PE as a mechanism for improving healthy habits and SWB in this population. Effectively, the combination of PEs developed herein were associated with physical benefits such as fat loss (especially visceral fat), improved lean muscle mass, improved metabolic efficiency, better physical fitness, and reduced risk of cardiovascular diseases (Church et al., 2010; Schwingshackl et al., 2014). The program can also contribute to social support and interaction development since such exercise programs are influenced by social elements (e.g., group class, coach, and teacher) that often impact loneliness and isolation (Ahn et al., 2024). The combined exercise program also has great influences on improving anxiety and depression, social dysfunction, loss of confidence, and general psychological wellbeing among patients with T2D (Gebeyehu et al., 2022).

According to Diener et al. (1985), global life satisfaction is a cognitive judgment of the quality of one’s own life. Hence, the benefits of PE can also extend to improved quality of life and consequently SWL. Effectively, exercise and SWL are positively linked since physically active people have higher levels of SWL than physically inactive people (Iwon et al., 2021). Otherwise, the affective dimension of SWB reflects the basic experience within events occurring in one’s life. There are two dimensions to the affective components of SWB, namely positive and negative affect. The positive affect or pleasant emotions are part of SWB because these emotions reflect individual reactions to events that show that life goes as desired (Diener et al., 1985), and more daily PA is associated with higher positive affect (Ludwig and Rauch, 2018). Exercise is also linked to the release of endorphins that act as painkillers and mood elevators to strengthen the feelings of wellbeing (Boecker et al., 2008). Exercise is known to help improve insulin sensitivity and glucose control, which can reduce the psychological stressors associated with T2D (Colberg et al., 2016). Moreover, exercise programs that include social interactions and peer support have been shown to result in higher levels of emotional wellbeing and positive affect in T2D subjects compared to those exercising alone (Paul et al., 2007). Since the T2D subjects reported more PA and healthy nutritional habits after intervention, the findings corroborate the results of previous studies (Colberg et al., 2016; Wing and Phelan, 2005). Beyond improving physical fitness that would allow people with T2D to be more physically active during their daily lives, the structured exercise programs provide an environment that encourages regular PA, making it easier to develop and maintain healthy habits (WHO, 2024a). Furthermore, being a part of a group can offer social support and motivation, which are essential for sustaining long-term behavioral changes (CDC, 2024). People with T2D who were involved in a positive feedback loop with nutrition emphasized that the combination of regular exercise and improved dietary habits created a synergistic effect, leading to sustained improvements in both PA levels and nutrition, especially in individuals with severe metabolic conditions (Dalle Grave et al., 2010).

Despite the encouraging results, the generalization of the results should be carefully considered given the main limitations of this study. The sample size limits generalizability and also makes it difficult to extend the results to larger populations with different sociodemographic characteristics. The present study design does not allow us to assess the results through a follow-up period, which is essential for evaluating the long-term effects of the observed results. A comparative analysis with a control group that is not involved in the same intervention is therefore essential to guarantee that the results are exclusively linked to the *Diabetes em Movimento* ^®^ program. This methodological limitation also restricts internal validity and compromises the capacity to securely affirm that the observed effects are derived exclusively from the intervention. Therefore, regarding the future directions of this work, we consider it essential that the study be developed with access to a larger sample group along with a follow-up period as well as the presence of a control group through a randomized process to strengthen the evidence.

## Conclusion

The main findings of study II suggest that people involved in the *Diabetes em Movimento* ^®^ program reported improved SWB and key lifestyle factors through significant increases in the positive affect, SWL, PA, and nutrition habits along with a notable decrease in the negative affect between the baseline and post-intervention period. The present results support evidence confirming the positive effects of PE through the *Diabetes em Movimento* ^®^ program to foster SWB and promote healthier lifestyle behaviors among T2D subjects.

## General conclusions and limitations

Based on the main findings of study I, we identified that the factorial structure of the *Fantastic Lifestyle* questionnaire (i.e., nine factors, 27 items) is a valid and reliable tool for assessing the lifestyle variables of the Portuguese population. Furthermore, we identified in study II that the implementation of the PE program *Diabetes em Movimento* ^®^ was beneficial for improving the positive affect, SWL, PA, sleep, stress, and nutritional behaviors significantly while notably diminishing the negative affect in our sample of people with T2D. As noted before, these results should be interpreted with some caution as our study did not have a control group and the sample was one of convenience. Although the present work can be classified as a pretest/posttest study without a control group, which would allow us to compare the measurements before and after the intervention, the lack of a control group and absence of sample randomization necessitates a somewhat cautious claim that the changes observed herein are exclusively attributable to the intervention. Nevertheless, these results can provide insights into the effectiveness of an intervention even though they are limited in terms of internal validity. We should therefore conduct further studies in the future to consolidate the results achieved in the present study, particularly by including control groups or applying sample randomization methods.

## Data Availability

The raw data supporting the conclusions of this article will be made available by the authors without undue reservation.

## References

[B1] Ahn J. Falk E. B. Kang Y. (2024). Relationships between physical activity and loneliness: a systematic review of intervention studies. Curr. Res. Behav. Sci. 6, 100141. 10.1016/j.crbeha.2023.100141

[B2] Amanat S. Ghahri S. Dianatinasab A. Fararouei M. Dianatinasab M. (2020). Exercise and type 2 diabetes. Adv. Exp. Med. Biol. 1228, 91–105. 10.1007/978-981-15-1792-1_6 32342452

[B65] American College of Sports Medicine (2022). ACSM’s Guidelines for Exercise Testing and Prescription (11th ed.). Wolters Kluwer. 10.1249/JSR.0b013e31829a68cf 23851406

[B3] Anderson R. J. Freedland K. E. Clouse R. E. Lustman P. J. (2001). The prevalence of comorbid depression in adults with diabetes: a meta-analysis. Diabetes Care 24 (6), 1069–1078. 10.2337/diacare.24.6.1069 11375373

[B4] Añez C. Reis R. S. Petroski E. L. (2008). Versão brasileira do questionário “estilo de vida fantástico”: tradução e validação para adultos jovens. Arq. Bras. Cardiol. 91, 102–109. 10.1590/S0066-782X2008001400006

[B5] Antunes R. Couto N. Vitorino A. Monteiro D. Marinho D. A. Cid L. (2020). Physical activity and affect of the elderly: contribution to the validation of the positive and negative affect schedule (PANAS) in the Portuguese population. J. Hum. Sport Exerc. 15 (2). 10.14198/jhse.2020.152.08

[B6] Antunes R. Couto N. Vitorino A. Monteiro D. Moutão J. M. Marinho D. (2019). Physical activity and satisfaction with the life of the elderly: contribution to the validation of satisfaction with life scale (SWLS) in the Portuguese population. Rev. Iberoam. Psicol. Del Ejerc. El Deporte 14, 24.

[B7] Boecker H. Sprenger T. Spilker M. E. Henriksen G. Koppenhoefer M. Wagner K. J. (2008). The runner’s high: opioidergic mechanisms in the human brain. Cereb. Cortex (New York, N.Y. 1991) 18 (11), 2523–2531. 10.1093/cercor/bhn013 18296435

[B8] Borg A. (1982). Psychophysical bases of perceived exertion. Med. Sci. Sports Exerc. 14 (5), 377–381. 10.1249/00005768-198205000-00012 7154893

[B9] Borg G. (1998). Borg’s Perceived Exertion and Pain Scales. Champaign: Human Kinetics.

[B10] Byrne B. M. (2016). Structural Equation Modeling with AMOS: Basic Concepts, Applications. 3rd ed. New York, NY: Routledge.

[B11] CDC (2024). Strategies for increasing physical activity. Phys. Act. Available online at: https://www.cdc.gov/physical-activity/php/public-health-strategy/index.html.

[B12] Chudyk A. Petrella R. J. (2011). Effects of exercise on cardiovascular risk factors in type 2 diabetes: a meta-analysis. Diabetes Care 34 (5), 1228–1237. 10.2337/dc10-1881 21525503 PMC3114506

[B13] Church T. S. Blair S. N. Cocreham S. Johannsen N. Johnson W. Kramer K. (2010). Effects of aerobic and resistance training on hemoglobin A _1c_ levels in patients with type 2 diabetes: a randomized controlled trial. JAMA 304 (20), 2253–2262. 10.1001/jama.2010.1710 21098771 PMC3174102

[B14] Cid L. Monteiro D. Teixeira D. S. Evmenenko A. Andrade A. Bento T. (2022). Assessment in sport and exercise psychology: considerations and recommendations for translation and validation of questionnaires. Front. Psychol. 13, 806176. 10.3389/fpsyg.2022.806176 35360588 PMC8963805

[B15] Cohen J. (1988). Statistical Power Analysis for the Behavioral Sciences. Hillsdale: Lawrence Erlbaum Associates.

[B16] Colberg S. R. Sigal R. J. Fernhall B. Regensteiner J. G. Blissmer B. J. Rubin R. R. (2010). Exercise and type 2 diabetes: the American college of sports medicine and the American diabetes association: joint position statement. Diabetes Care 33 (12), e147–e167. 10.2337/dc10-9990 21115758 PMC2992225

[B17] Colberg S. R. Sigal R. J. Yardley J. E. Riddell M. C. Dunstan D. W. Dempsey P. C. (2016). Physical activity/exercise and diabetes: a position statement of the American diabetes association. Diabetes Care 39 (11), 2065–2079. 10.2337/dc16-1728 27926890 PMC6908414

[B18] Dalle Grave R. Calugi S. Centis E. Marzocchi R. El Ghoch M. Marchesini G. (2010). Lifestyle modification in the management of the metabolic syndrome: achievements and challenges. Diabetes Metabolic Syndrome Obes. Targets Ther. 3, 373–385. 10.2147/DMSOTT.S13860 21437107 PMC3047997

[B19] Darviri C. Alexopoulos E. C. Artemiadis A. K. Tigani X. Kraniotou C. Darvyri P. (2014). The healthy lifestyle and personal control questionnaire (HLPCQ): a novel tool for assessing self-empowerment through a constellation of daily activities. BMC Public Health 14 (1), 995. 10.1186/1471-2458-14-995 25253039 PMC4192765

[B66] Denis D. J. (2019). SPSS data analysis for univariate, bivariate, and multivariate statistics. Hoboken, NJ: John Wiley & Sons.

[B20] Diener E. Emmons R. A. Larsen R. J. Griffin S. (1985). The satisfaction with life scale. J. Personality Assess. 49 (1), 71–75. 10.1207/s15327752jpa4901_13 16367493

[B21] Dinzeo T. J. Thayasivam U. Sledjeski E. M. (2013). The development of the lifestyle and habits questionnaire-brief version: relationship to quality of life and stress in college students. Prev. Sci. 15 (1), 103–114. 10.1007/s11121-013-0370-1 23417669

[B22] Dong W. Xiao-hui X. Xian-bo W. (2012). The healthy lifestyle scale for university students: development and psychometric testing. Aust. J. Prim. Health 18 (4), 339–345. 10.1071/PY11107 22950906

[B23] European Union (2022). Special eurobarometer 525: sport and physical activity. Available online at: https://europa.eu/eurobarometer/surveys/detail/2668.

[B24] Faul F. Erdfelder E. Buchner A. Lang A.-G. (2009). Statistical power analyses using G*Power 3.1: tests for correlation and regression analyses. Behav. Res. Methods 41 (4), 1149–1160. 10.3758/BRM.41.4.1149 19897823

[B25] Ferreira J. P. Duarte-Mendes P. Teixeira A. M. Silva F. M. (2022). Effects of combined training on metabolic profile, lung function, stress and quality of life in sedentary adults: a study protocol for a randomized controlled trial. PLoS One 17 (2), e0263455. 10.1371/journal.pone.0263455 35113957 PMC8812960

[B26] Fisher L. Glasgow russel Strycker L. (2010). The relationship between diabetes distress and clinical depression with glycemic control among patients with type 2 diabetes. Diabetes Care 33 (5), 1034–1036. 10.2337/dc09-2175 20150291 PMC2858170

[B27] Fonseca A. M. Paula Brito A. (2005). A questão da adaptação transcultural de instrumentos para avaliação psicológica em contextos desportivos nacionais—O caso do Task and Ego Orientation in Sport Questionnaire (TEOSQ). The issue of the cross-cultural adjustment of instruments for psychological evaluation in national sport contexts—The case of the Task and Ego Orientation in Sport Questionnaire (TEOSQ). Psychologica 39, 95–118.

[B28] Fornell C. Larcker D. F. (1981). Evaluating structural equation models with unobservable variables and measurement error. J. Mark. Res. 18 (1), 39. 10.2307/3151312

[B29] Gebeyehu M. Legesse K. Mondal S. Abdulkadir M. Bekelle Z. Molla A. (2022). Effects of structured exercises on selected psychological domains in individuals with type 2 diabetes mellitus in southern Ethiopia institution-based study. BMC Sports Sci. Med. Rehabilitation 14, 181. 10.1186/s13102-022-00574-3 36224647 PMC9558398

[B30] Hair J. F. Babin B. J. Anderson R. E. Black W. C. (2019). Multivariate data analysis. 8th ed. England: Pearson Prentice.

[B31] Hajos T. R. S. Pouwer F. de Grooth R. Holleman F. Twisk J. W. R. Diamant M. (2012). The longitudinal association between glycaemic control and health-related quality of life following insulin therapy optimisation in type 2 diabetes patients. A prospective observational study in secondary care. Qual. Life Res. Int. J. Qual. Life Aspects Treat. Care Rehabilitation 21 (8), 1359–1365. 10.1007/s11136-011-0051-0 22065281 PMC3438404

[B32] Hayashino Y. Jackson J. L. Fukumori N. Nakamura F. Fukuhara S. (2012). Effects of supervised exercise on lipid profiles and blood pressure control in people with type 2 diabetes mellitus: a meta-analysis of randomized controlled trials. Diabetes Res. Clin. Pract. 98 (3), 349–360. 10.1016/j.diabres.2012.10.004 23116535

[B33] Hill M. M. Hill A. (2008). Investigação por Questionário | Edições Sílabo. 2nd ed. Edições Sílabo Ltda.

[B34] Ho D. E. Imai K. King G. Stuart E. A. (2007). Matching as nonparametric preprocessing for reducing model dependence in parametric causal inference. Polit. Anal. 15 (3), 199–236. 10.1093/pan/mpl013

[B35] IDF (2021). IDF diabetes atlas. 10th ed. Available online at: https://diabetesatlas.org/atlas/tenth-edition/.

[B36] Iwon K. Skibinska J. Jasielska D. Kalwarczyk S. (2021). Elevating subjective well-being through physical exercises: an intervention study. Front. Psychol. 12, 702678. 10.3389/fpsyg.2021.702678 34975608 PMC8719442

[B37] Kline R. B. (2016). Principles and practice of structural equation modeling. 4th ed. New York, NY: The Guilford Press.

[B38] Knowler W. Barrett-Connor E. Fowler S. Hamman R. Lachin J. Walker E. (2002). Reduction in the incidence of type 2 diabetes with lifestyle intervention or metformin. N. Engl. J. Med. 346 (6), 393–403. 10.1056/NEJMoa012512 11832527 PMC1370926

[B39] Lloyd A. Sawyer W. Hopkinson P. (2001). Impact of long-term complications on quality of life in patients with type 2 diabetes not using insulin. Value Health J. Int. Soc. Pharmacoecon. Outcomes Res. 4 (5), 392–400. 10.1046/j.1524-4733.2001.45029.x 11705130

[B40] López-Carmona J. M. Rodríguez-Moctezuma R. Munguía-Miranda C. Hernández-Santiago J. L. Torre E. C. de la (2000). Validez y fiabilidad del instrumento «FANTASTIC» para medir el estilo de vida en pacientes mexicanos con hipertensión arterial. Atencion Primaria 26 (8), 542–549. 10.1016/S0212-6567(00)78719-1 11149187 PMC7679574

[B67] Ludwig K. Rauch W. A. (2018). Associations between physical activity, positive affect, and self-regulation during preschoolers’ everyday lives. Mental Health and Physical Activity, 15, 63–70. 10.1016/j.mhpa.2018.07.002

[B41] Mendes R. Sousa N. Almeida A. Subtil P. Guedes-Marques F. Reis V. M. (2016). Exercise prescription for patients with type 2 diabetes-a synthesis of international recommendations: narrative review. Br. J. Sports Med. 50 (22), 1379–1381. 10.1136/bjsports-2015-094895 26719499

[B42] Mendes R. Sousa N. Reis V. Themudo Barata J. (2017). Implementing low-cost, community-based exercise programs for middle-aged and older patients with type 2 diabetes: what are the benefits for glycemic control and cardiovascular risk? Int J Environ Res Public Health. 4 (4), 18–20. 10.3390/ijerph14091057 PMC5615594 28902144

[B43] Mendes R. Sousa N. Reis V. M. Barata J. L. T. (2016). Programa Exercício Diabetes Tipo 2 6 (2), 62–70.

[B44] Moctezuma R. R. Carmon J. Miranda C. Santiago J. Bermúdez M. (2003). Validez y consistencia del instrumento FANTASTIC para medir estilo de vida en diabéticos.

[B68] Nelson M. E. Rejeski W. J. Blair S. N. Duncan P. W. Judge J. O. King A. C. Macera C. A. Castaneda-Sceppa C. (2007). Physical activity and public health in older adults: recommendation from the American College of Sports Medicine and the American Heart Association. Med Sci Sports Exerc. 39 (8), 1435–1445. 10.1249/mss.0b013e3180616aa2 17762378

[B45] Nevitt J. Hancock G. R. (2001). Performance of bootstrapping approaches to model test statistics and parameter standard error estimation in structural equation modeling. Struct. Equ. Model. 8 (3), 353–377. 10.1207/S15328007SEM0803_2

[B46] Nunnally J. Bernstein I. (1994). Psychometric theory. 3rd ed. McGraw-Hill, Inc.

[B47] Oftedal S. Vandelanotte C. Duncan M. J. (2019). Patterns of diet, physical activity, sitting and sleep are associated with socio-demographic, behavioural, and health-risk indicators in adults. Int. J. Environ. Res. Public Health 16 (13), 2375. 10.3390/ijerph16132375 31277386 PMC6651368

[B48] Oliveira C. Simões M. Carvalho J. Ribeiro J. (2012). Combined exercise for people with type 2 diabetes mellitus: a systematic review. Diabetes Res. Clin. Pract. 98 (2), 187–198. 10.1016/j.diabres.2012.08.004 22981711

[B49] Paul G. M. Smith S. M. Whitford D. L. O’Shea E. O’Kelly F. O’Dowd T. (2007). Peer support in type 2 diabetes: a randomised controlled trial in primary care with parallel economic and qualitative analyses: pilot study and protocol. BMC Fam. Pract. 8, 45. 10.1186/1471-2296-8-45 17672892 PMC1950508

[B50] Plotnikoff R. C. Costigan S. A. Karunamuni N. D. Lubans D. R. (2013). Community-based physical activity interventions for treatment of type 2 diabetes: a systematic review with meta-analysis. Front. Endocrinol. 4, 3. 10.3389/fendo.2013.00003 23372566 PMC3557414

[B51] Ramírez-Vélez Agredo A. (2012). Fiabilidad y validez del instrumento “Fantástico” para medir el estilo de vida en adultos colombianos. Rev. Saúde Pública 14 (2), 226–237. 10.1590/S0124-00642012000200004 23250366

[B52] Raykov T. (1997). Estimation of composite reliability for congeneric measures. Appl. Psychol. Meas. 21 (2), 173–184. 10.1177/01466216970212006

[B53] Sazlina S.-G. Browning C. Yasin S. (2013). Interventions to promote physical activity in older people with type 2 diabetes mellitus: a systematic review. Front. Public Health 1, 71. 10.3389/fpubh.2013.00071 24392445 PMC3870318

[B54] Schwingshackl L. Missbach B. Dias S. König J. Hoffmann G. (2014). Impact of different training modalities on glycaemic control and blood lipids in patients with type 2 diabetes: a systematic review and network meta-analysis. Diabetologia 57 (9), 1789–1797. 10.1007/s00125-014-3303-z 24996616

[B55] Silva A. Brito I. D. S. Amado J. M. D. C. (2014). Translation, adaptation and validation of the fantastic lifestyle assessment questionnaire with students in higher education. Ciência & Saúde Coletiva 19 (6), 1901–1909. 10.1590/1413-81232014196.04822013 24897489

[B56] Silva F. M. Duarte-Mendes P. Teixeira A. M. Soares C. M. Ferreira J. P. (2024). The effects of combined exercise training on glucose metabolism and inflammatory markers in sedentary adults: a systematic review and meta-analysis. Sci. Rep. 14 (1), 1936. 10.1038/s41598-024-51832-y 38253590 PMC10803738

[B57] Umpierre D. Ribeiro P. A. B. Kramer C. K. Leitão C. B. Zucatti A. T. N. Azevedo M. J. (2011). Physical activity advice only or structured exercise training and association with HbA1c levels in type 2 diabetes: a systematic review and meta-analysis. JAMA 305 (17), 1790–1799. 10.1001/jama.2011.576 21540423

[B58] WHO (1999). Healthy living: what is a healthy lifestyle? No. EUR/ICP/LVNG010702. Available online at: https://iris.who.int/handle/10665/108180.

[B59] WHO (2009). Interventions on diet and physical activity: what works: summary report. Available online at: https://www.who.int/publications/i/item/interventions-on-diet-and-physical-activity-what-works-summary-report. 24432437

[B60] WHO (2024a). Physical activity. Available online at: https://www.who.int/news-room/fact-sheets/detail/physical-activity.

[B61] WHO (2024b). Self-care for health and well-being. Available online at: https://www.who.int/news-room/fact-sheets/detail/self-care-health-interventions.

[B62] Wilson D. M. C. Nielsen E. Ciliska D. (1984). Lifestyle assessment: testing the FANTASTIC instrument. Can. Fam. Physician 30, 1863–1866.

[B63] Wing R. R. Phelan S. (2005). Long-term weight loss maintenance. Am. J. Clin. Nutr. 82 (1), 222S-225S–225S. 10.1093/ajcn/82.1.222S 16002825

[B64] Worthington R. L. Whittaker T. A. (2006). Scale development research: a content analysis and recommendations for best practices. Couns. Psychol. 34 (6), 806–838. 10.1177/0011000006288127

